# How do small rural primary health care services sustain themselves in a constantly changing health system environment?

**DOI:** 10.1186/1472-6963-12-81

**Published:** 2012-03-26

**Authors:** Penny Buykx, John S Humphreys, Rachel Tham, Leigh Kinsman, John Wakerman, Adel Asaid, Kathy Tuohey

**Affiliations:** 1 Monash University School of Rural Health, PO Box 666, Bendigo, VIC 3552, Australia; 2 Centre for Remote Health, A joint Centre of Flinders University & Charles Darwin University, PO Box 4066, Alice Springs, NT 0870, Australia; 3 Elmore Primary Health Service, 46-48 Jeffrey Street, Elmore, VIC 3558, Australia; 4 Centre of Research Excellence in Rural and Remote Primary Health Care, Bendigo, Australia

## Abstract

**Background:**

The ability to sustain comprehensive primary health care (PHC) services in the face of change is crucial to the health of rural communities. This paper illustrates how one service has proactively managed change to remain sustainable.

**Methods:**

A 6-year longitudinal evaluation of the Elmore Primary Health Service (EPHS) located in rural Victoria, Australia, is currently underway, examining the performance, quality and sustainability of the service. Threats to, and enablers of, sustainability have been identified from evaluation data (audit of service indicators, community surveys, key stakeholder interviews and focus groups) and our own observations. These are mapped against an overarching framework of service sustainability requirements: workforce organisation and supply; funding; governance, management and leadership; service linkages; and infrastructure.

**Results:**

Four years into the evaluation, the evidence indicates EPHS has responded effectively to external and internal changes to ensure viability. The specific steps taken by the service to address risks and capitalise on opportunities are identified.

**Conclusions:**

This evaluation highlights lessons for health service providers, policymakers, consumers and researchers about the importance of ongoing monitoring of sentinel service indicators; being attentive to changes that have an impact on sustainability; maintaining community involvement; and succession planning.

## Background

Under the current Australian health reform agenda, a high priority has been given to the provision of integrated, comprehensive PHC services that are sustainable and responsive to community needs [1]. As PHC services are usually the first point of contact with the health service system, the loss of such services contributes to diminished access to health care and poorer health outcomes, especially in rural and remote communities [2]. Despite research advocating innovative sustainable models [3,4], the problems of maintaining quality PHC services in isolated communities characterised by small, dispersed populations remain of paramount concern for communities and providers.

This paper describes how one small rural PHC service is adapting to significant ongoing external (both political and health system environment) and internal (such as workforce) changes to ensure its sustainability. Changes are constantly occurring across all areas of PHC service operation, including: workforce supply (the difficulty of recruitment to, and retention in, rural areas); increased need for linkages with other health care providers (because the rising chronic disease burden necessitates coordination of care between services for a greater number of patients); infrastructure demands (due to technological advances in medical information systems, equipment and procedures); and leadership and management accountability requirements (to address increasing community expectations and participation, and funding requirements). In addition, health service managers are required to address these changes within an environment characterised by increasing demand for health care on the one hand and severe fiscal constraint on the other.

Most research examining rural health service sustainability comprises descriptive case-studies undertaken at a single point in time, many of which suggest that many rural health services do not adapt easily to emerging community health needs and the demands of a changing external environment. This longitudinal study (2008-14) provides a unique opportunity to investigate how one service is responding to significant changes which affect service sustainability. Understanding how to respond to change both proactively and reactively enables rural PHC services to cope with the impact of economic, social and political processes and provides crucial evidence for planning.

### How does change affect rural health service sustainability?

Health service sustainability is a key dimension of Australian health policy [5], including rural health service policies [6-9]. 'Sustainability' refers to the ability of a health service to provide ongoing access to appropriate quality care in a cost-efficient and health-effective manner [10] and implies a capacity to persist in, and adapt to, a changing environment. A viable health service also has the potential to positively influence the broader sustainability of its local community [11]. The need for sustainable health services is particularly acute in small rural and remote communities where the challenges associated with delivering a comprehensive range of primary care services are greatest [2].

Threats to rural health service sustainability include small population sizes and associated lack of economies of scale, difficulty in maintaining an adequate workforce, poor management structures, geographic isolation, and the demands of primary, acute and chronic care needs in socio-economically disadvantaged populations [12]. These problems are exacerbated by out-migration, ageing population, and changing consumer expectations. Changes in government policy have a major impact on funding or organisational arrangements. For example, in Australia, government adoption of a new rural classification system has altered the eligibility of rural health doctors for rural medical incentive payments [13], while the effect of introducing PHC networks (i.e. Medicare Locals) on individual PHC services remains unclear [14], despite their mandate for population needs-based service provision under this system.

In order to assess and monitor the impact of change on sustainability, it is important to adopt a systems approach [10]. That is, we focus on how individual components, both within and external to the health service, contribute to the efficient and effective functioning of the whole organisation. In this study the enablers of, and threats to, sustainability are considered within a comprehensive framework of PHC service requirements [15,16]. These inter-dependent sustainability requirements (workforce organisation and supply; funding; governance, management and leadership; service linkages; and infrastructure [15,17]) have been validated previously across a range of different primary health service models in different rural and remote contexts [16]. Sustainable PHC services also depend on several external environmental enablers, including a supportive policy environment, clearly-articulated Federal-State governments' roles and responsibilities, and strong community involvement. This study helps to redress the knowledge gap about how rural health services adapt to these important external changes in order to ensure service sustainability.

## Methods

Elmore (population 700) is located 170 km from Melbourne in central Victoria, Australia. The Elmore Primary Health Service (EPHS) longitudinal evaluation (2008-14) was initiated with the specific purpose of monitoring (i) the provision of appropriate, quality health care to the Elmore community and surrounding region, (ii) the impact of ongoing changes on service sustainability, and (iii) community satisfaction with, and utilisation of, the health service. Details of the methods used have been published previously [18]. Evaluation data include an annual audit of sentinel service indicators, community surveys (conducted in 2008 and 2010), in-depth interviews with key stakeholders, and focus groups [18,19]. In this paper we draw on those data relevant to service sustainability and our own detailed observations to synthesise a narrative about how the service is continuing to meet local health care needs, despite significant internal and external changes that threaten its sustainability.

### Ethics

The study obtained approval from Monash University Human Research Ethics Committee (CF08/0419- 2008000176; CF08/0238 - 2008000089; CF08/2434 - 2008001256; CF10/2540 - 20100001423).

## Results

### How did Elmore develop a successful PHC service?

Historically, Elmore maintained its own hospital and resident doctor. In 1994, however, the hospital was closed as part of a state government program of health service rationalisation and centralisation. Shortly thereafter the doctor left. The remaining health services (i.e. the district nurse and maternal and child health nurse) were amalgamated with the nearby Rochester Hospital by the health authority to form the Rochester and Elmore District Health Service. Residents then had to travel to Rochester (17 km NE) or Bendigo (45 km SW) to access other health services.

Following a period of negotiation between the regional health authority, the community, and key health stakeholders, the EPHS was formed in 2004. The timetable and factors underpinning the successful establishment of EPHS, including a detailed discussion of its structure and function, have been reported elsewhere [18,20]. By 2009-10 there were eight general practitioners (GPs), three nurses, three allied health practitioners and nine administrative staff working in Elmore (respective full time equivalents approximately 2.7, 0.8, 0.5 and 5.3) serving a catchment population of 2760 patients from the Elmore township and its hinterland attending the service at least once during the year [21]. Our research identified four key success factors underpinning the development of this comprehensive, single-point-of-entry PHC service model.

#### i. **Community engagement**

The mandated Rochester and Elmore District Health Service amalgamation was poorly received by Elmore residents. The community felt that the consultation process lacked transparency, there was a decline in the standard and continuity of service delivery, and they had lost their autonomy and the health infrastructure in which they had invested. Additionally, the community was weakened economically from the loss of local employment and population. However, this discontent proved to be the most important catalyst for action. In 1997 community volunteers formed the Elmore Working Group which aimed to regain community control over health services, develop an integrated model of health care, and recruit and retain doctors. This group was instrumental in engaging and maintaining the community in the early development and growth of EPHS.

#### ii. **Strong leadership and committed champions with a vision**

In 1999 the Elmore Working Group recruited an international medical graduate (IMG) as a solo general practitioner (GP) who established the Elmore Medical Practice (EMP) in the former hospital building. A community health nurse and practice manager employed by Rochester and Elmore District Health Service were co-located with EMP but were not part of the practice. This small group recognised the importance of a genuine PHC approach and worked together with the community and Bendigo Community Health Services (BCHS) to develop an integrated PHC organisation. This was crucial in gaining community ownership and participation [20].

#### iii. **Strategic relationship building**

To help realise their vision, the Elmore Working Group invited the state government Department of Human Services (DHS) to facilitate community consultation meetings to investigate possible health service delivery models to suit the needs of the Elmore community. Through this process the community determined their desire for BCHS to provide community health services in partnership with EMP, resulting in a partnership arrangement designed to ensure local availability of a comprehensive range of health care services. A public-private funding model was developed for a comprehensive, integrated health service. The model combined fee-for-service Medicare-based income deriving from the private medical practice with public funding from services provided by BCHS. This model provided a very strategic approach to optimise use of scarce health staff while at the same time maximising community access to a comprehensive range of health care services.

#### iv. **Health service linkages**

Once the Elmore community endorsed this integrated service model, BCHS and EMP reviewed existing health service models for small rural communities. Models were matched against the community's expressed needs, and potential risks and benefits considered. Elmore now has a single-point-of-entry comprehensive PHC service (i.e. EPHS) which provides medical services, including after-hours emergency care, together with community health services (e.g. community nursing, health promotion, disease prevention and allied health services).

### How is the EPHS sustaining itself in the face of significant changes?

Currently EPHS is performing well across the spectrum of PHC (i.e. health promotion, early intervention, acute care and treatment of chronic diseases) with evidence of high levels of community satisfaction [22]. Local residents perceive EPHS to contribute to the growth and development of the town through the provision of health services and other community activities. The EPHS has also provided significant local employment and encouraged other related businesses (such as a pharmacy), to be established in the town.

As with many rural and remote communities, a key question is how has EPHS been able to sustain itself and how long will this success last? Since the service evaluation commenced in 2008, numerous significant health system policy and legislative changes have affected the operation and sustainability of EPHS. Changes that have threatened the sustainability of EPHS and the response of the service to these are shown in detail in Table 1. These are grouped according to previously identified key sustainability requirements [16].

**Table 1:** Steps taken by EPHS to address external and internal threats to service sustainability

Core health service sustainability requirement	Threats to service sustainability	Impact of threats on sustainability	Elmore health service responses and outcomes
***Addressing EXTERNAL threats to environmental enablers***			

**Supportive policy environment**	Changes in IMG legislation/regulations	• Recruitment and appointment process for IMGs is more difficult	• Targeted recruitment of potential doctors by EPHS staff • Greater dependence on assistance of Rural Workforce Agency Victoria (RWAV)
	• Changes in funding arrangements (e.g. after-hours services)	• Affects total amount and mix of funding available to service	• Broaden income base through more education and training, research and incentive funding
	• Changes in government funding schedule and service indicators	• Attempts by government to reduce the 'red-tape' requirements have complicated service performance monitoring and associated quality improvement	• Strengthen link with research evaluation team to identify and maintain sentinel indicators for measuring performance

**Clearly-articulated Federal-State roles and responsibilities**	• Announcement of nation-wide orientation to PHC models and organisations (Medicare Locals)	• Implementation distracting service staff and workforce agencies from 'core business'	• Service is positioning itself with key agencies and authorities to maintain its role and visibility in new regional organisational arrangements

**Strong community involvement**	• Changing demography; impact of natural disasters (floods, bushfires) in the catchment area	• Population change due to ageing and in- and out-migration make it difficult to engage broad population in early intervention and results in need for different services	• Establishment of a single-point-of-entry to comprehensive PHC ensures access to the range of integrated services providing acute and chronic care, health promotion and disease prevention • Regular consultation with community about service changes

***Addressing INTERNAL threats to service sustainability requirements***			

**Workforce supply and mix**	• Rapid expansion of EPHS catchment (i.e. into surrounding regions: 'hub-and-spoke' model of visiting services and establishment of permanent services in surrounding region)	• Risk of expansion beyond workforce capability and service capacity, high cost of ongoing recruitment	• Targeted recruitment ensures prospective staff are well-matched to service • Use of one doctor to provide locum relief across all sites
	• Ongoing dependence on IMGs	• Risk of short length of stay and need to re-recruit as IMGs relocate to metropolitan areas for cultural and family reasons	Staff retention maximised by: • Good matching of recruits to the service • Strong supervision and support for continuing professional development • Capitalising on the full range of workforce incentives • Critical mass of GPs means after-hours work is not too demanding and enables part-time work • Multidisciplinary teamwork reduces isolation and workload
	• Growth of GP 'superclinic' in nearby large regional centre [23]	• May provide a more attractive alternative practice location for doctors	• Existing service maintains comprehensive whole-of-patient and community care activities that provide many professional opportunities and career satisfaction
	• Older staff seek retirement or career change	• Need for pro-active succession planning to minimise impact of loss of experienced staff	• Links to Monash University and RWAV as a teaching practice for medical students and registrars • Proactive succession planning

**i. Linkages**	• New leadership and change within partner organisations and government authorities	• Established relationships can be threatened by new arrangements that do not meet local needs and the complex public-private mix of services, ownership and investment arrangements	• Close collaboration with partners and ongoing involvement with established research team

**ii. Infrastructure**	• Infrastructure renewal required to accommodate organisational change and additional services	• Remodelling existing 'hospital' infrastructure can result in perceived 'loss' of services by some community residents	• Capitalising on infrastructure grants (e.g. new payment facilities, remodelling of infrastructure and 24/7 emergency care)

**iii. Funding**	• Dependence on fee-for-service funding and high level of bulk-billing • Changes to funding arrangements for after-hours service	• Diversification of financial sources required to ensure viability (i.e. total funding and blended-payment funding)	• Service capitalises on full range of financial incentives on offer (e.g. additional funding for after-hours service)
	Alternative services available in surrounding communities	• Patient attrition (e.g. following "usual doctor" to another practice, minimising the distance travelled by 'one-stop-shopping' in larger centres) affects income stream	• The comprehensive integrated range of services minimises patient leakage and maximises practice income

**iv. Governance leadership and management**	• Leadership changes (e.g. principal GP expands practice to other towns; new Chief Executive Officer recruited to key partner organisation [BCHS])	• When organisational leaders reduce or withdraw their services, the community may experience a sense of "loss" and perceive the quality of the services to have declined • Potentially weakened relationships between key partners	• The need for pro-active leadership succession planning within the health service is recognised • Mentoring new staff for clinical leadership roles • Practice manager shares expertise with and devolves responsibility to other administrative staff • Use new developments (e.g. building works) as an opportunity to revitalise relationship with key partners and extend opportunity for joint service provision

The sustainability and growth of EPHS has occurred not just because of any one key success factor, but rather because the service has pro-actively seized opportunities to strengthen the service and implemented practical responses to risks and threats. For example, consistent with its vision to meet as many community health needs as possible through local service provision, EPHS successfully sought more than half a million dollars in funding through competitive infrastructure grants to enhance and expand the service premises, thereby enabling service expansion and better integration. In a similarly pro-active way, the service has undertaken local community needs analysis in relation to its aged care. In contrast, the top-down imposition of changes to funding arrangements has affected the economic viability of the service, which has reacted by broadening its funding streams. Service activities are now constantly monitored to ensure quality improvement, efficiency and effectiveness. Capacity to undertake such monitoring has been built through partnership with the Monash University research team and the local Division of General Practice. This rigorous evaluation based on key sustainability requirements provides a valuable means of documenting how small PHC services address threats to their sustainability.

### Implications for policy, practice and research

Longitudinal rural health service evaluations such as this one help inform our understanding of why some rural health services succeed while others succumb to the impact of change, providing valuable insights for policy makers, service providers and researchers. Here we highlight sustainability issues related to ongoing evaluation, community participation, funding, succession planning and knowledge translation.

#### Lessons for policy-makers, service providers and consumers

• *Importance of monitoring service performance to ensure appropriate, high quality care: *Validated, evidence-based performance indicators are required to measure sentinel aspects of service provision. However, compliance with best practice can only be assessed if there is a system to enable monitoring of activities over time against performance benchmarks. Quality improvements not only benefit patient care, service efficiency and professional satisfaction, but can also increase the funding coming into the health service.

• *Remaining alert to environmental enablers: *The nature, scale and speed of changes at both macro and micro levels requires continuous assessment of their direct and indirect impacts upon the performance and sustainability of the local service.

• *Community participation: *Just as community participation was vital in the evolution and acceptance of a comprehensive PHC service, so ongoing community involvement is required to sustain a service geared towards community needs and in managing the impact of change. It is essential that information is shared beyond the service about what changes are occurring, why, and their likely effects in order to avoid a perception of loss of services or decline in the quality of care as the service evolves and adapts to new external requirements.

• *Succession planning: *Community leadership succession planning is essential to maintain dialogue between the community and the health service. For example, some of the early community leaders no longer play such a central role in advocating for the health needs of the community and in informing the community about what is happening in the service - new leaders are now required. Organisational succession planning is vital. Staff recruitment and development processes should ensure leadership skills (e.g. vision, good communication, initiative) exist within the staff group.

• *Evaluation funding: *Longitudinal evaluations require (i) appropriate funding to make them happen, and (ii) an anchor person within the service. While monitoring service performance should be integral to health service activity, the reality is that significant time, skills and expertise are required to establish an appropriate methodology, data collection and analysis framework for a specific service. Data collection and feedback for rigorous health service evaluation is a process rather than a one-off activity. Few services have the additional capacity or skills to undertake such monitoring. The timeliness and consistency of data collection activities can be improved by identifying and training a suitable person within the service to undertake the evaluation tasks (while acknowledging the need for succession planning for these tasks). External funding for evaluation should therefore make appropriate recompense for service time and include provision for capacity building.

#### Lessons for researchers undertaking collaborative health service evaluation

• *Demonstrable benefits to both parties: *Evaluations risk being intrusive and onerous unless they are developed collaboratively and there is an explicit feedback loop. Benefits may include simultaneously the capacity to bring about quality improvement for the health service, and the generation of new knowledge for academics.

• *Capacity building, communication and commitment: *Health service evaluation should be embedded as an integral aspect of a service [24]: long-term health service evaluations are at risk if they are overly dependent on a single person and not well-founded among all health service staff. Excellent ongoing communication between the evaluation team, the staff and the consumers of the health service is therefore vital. Regular meetings, newsletters, feedback presentations, and reference groups all contribute to a lasting relationship that provides security to the study.

• *Knowledge translation: *Rigorous evaluation can constitute good research. Not only can it generate new knowledge, but often more importantly these insights bring applied benefits to the health care available to communities; contribute to better and more viable health care services; and assist policy-makers to respond with appropriate and effective program support and interventions.

The research resources available through the collaboration between Monash University and EPHS have contributed to ongoing rigorous service evaluation and quality improvement are not commonly available to other rural health services. Partnership between a PHC service and a university has also previously been shown to have a positive impact on workforce recruitment and retention [25], an important aspect of sustainability. The importance of such university-community partnerships is only likely to increase. For example, the recent introduction of PHC networks in Australia, intended to improve planning for and co-ordination of PHC service delivery at a local population level, will require each 'Medicare Local' to undertake population-based needs assessments and to fulfil performance monitoring and reporting requirements. The capacity to self-evaluate and quality improve are integral to service planning and policy, and where that capacity is limited, these PHC organisations may choose to partner with organisations such as universities to provide the relevant expertise.

## Conclusion

While the longitudinal evaluation of the EPHS has only been underway for four years, it is already evident that EPHS is exemplary compared with many small rural community health services that have struggled to serve their communities adequately. Demographic, economic and political change is inevitable, and rural health services need to respond to its impact, regardless of whether it emanates from the macro- or micro-scale. In this regard, the EPHS model has demonstrated its capacity be proactive rather than simply reactive. It has done this through aligning its approach with government policies, ongoing monitoring of its service performance, maintaining strong engagement with the local community, maximising opportunities for alternative funding, initiating workforce succession planning and actively partnering research evaluation designed to ensure quality improvement. Policy makers, service providers, consumers and researchers can all play a significant role in ensuring that rural PHC services can adapt to, and benefit from, macro-scale social, economic and political changes, so that services continue to provide appropriate, high quality care to meet the health needs of the community.

## Abbreviations

BCHS: Bendigo community health services; DHS: Department of human services; EMP: Elmore medical practice; EPHS: Elmore primary health service; GP: General practitioner; IMG: International medical graduate; PHC: Primary health care.

## Competing interests

The authors declare that they have no competing interests.

## Authors' contributions

PB obtained ethics approval, collected and analysed data, and drafted and revised the manuscript. JSH and RT conceived and developed the design of the study, obtained ethics approval, collected and analysed data, and drafted and revised the manuscript. LK conceived and developed the design of the study, obtained ethics approval, and revised the manuscript. JW was the co-developer of the framework used in this study and contributed to the revision of the manuscript. AA and KT contributed to the conception and design of the study and revised the manuscript. All authors read and approved the final manuscript.

## Pre-publication history

The pre-publication history for this paper can be accessed here:

http://www.biomedcentral.com/1472-6963/12/81/prepub
